# Introductory Lecture to the Students of the Medical Department of the University of Buffalo, Delivered November 4th, 1868

**Published:** 1868-11

**Authors:** Sandford Eastman

**Affiliations:** Professor of Anatomy and Clinical Surgery


BUFFALO

### Medical and Surgical Journal.

VOL. VIII. NOVEMBER, 1868. No. 4.

### Original Communications.

ART. I.—*Introductory Lecture to the Students of the Medical Department of the University of Buffalo, delivered November 4th,* 1868. BY SANDFORD EASTMAN, M.D., *Professor of Anatomy and Clinical Surgery.*

GENTLEMEN:—In accordance with the general custom, and in compliance with the designation of our most worthy Dean, it is my agreeable duty and pleasure to address you on this interesting occasion, and offer you words of kindly greeting and hearty cheer; to extend to you the right hand of fellowship upon your advent in these scientific halls, and upon the threshold of your career as students in medicine. Some of you have occupied these seats on former occasions, and have imbibed from the fountain of knowledge here opened to you, such truths and facts as were in our power to communicate. Others are here for the first time, to compare what we teach with that discoursed upon in other medical schools, while some are here who have never attended any course of lectures, and perhaps have but just commenced the study of medicine, and may not, forsooth, have opened a medical book before entering these halls. To each and all I am commissioned to offer a cordial welcome, not only to the medical department of the University of Buffalo, and the clinical advantages afforded by the hospitals of our city, but also to the labors and incidents of students in medicine—for we are all *students.* Those of us who have assumed the duties of teachers in these halls are not forgetful of the fact that we were not long ago novices in our science and art, and we know full well that we must study and work, and study too with our work, to keep pace with the rapid progress that is being made in every department of medicine and surgery. The student or physician who is content with the curriculum of the schools of our pupilage, twenty or twenty-five years ago, will find himself outstripped both in theory and practice by the earnest and energetic student of the present day. I have not the time, neither is this the occasion, to note the great advance that has been made in the diagnosis and treatment of disease within the last quarter of a century. It ill becomes one who wants to encourage the student in the very commencement of his pupilage, to hold up the great store-house of knowledge, and assure him that all this he must be cognizant of before he can safely practice the healing art. No discouragement should be given the inquiring mind in its investigation and search for truth. Day by day will convince the vainest and most conceited student that he knows but little compared with what he does not know; and if he does not realize the fact before he graduates, it will come up before him in all its stupendous proportions ere he has long been striving with his professional brethren for success and eminence. The student on leaving college, upon the completion of his classical and literary course, is apt to think that he knows it *all;* that what he does not know is not worth knowing, but each recurring year more and more convinces him of the error of his boyhood, and he finds with each decade of his life that more remains to be learned than he has yet acquired. If the mantle of learning and wisdom, gotten by three-score years of patient labor and assiduous toil in the profession, could fall upon some of you, and you should be spared for the same period to extend your observations and researches for truth, you might then unfold and discover important facts hitherto hidden from mortal ken. But such learning must be acquired by you *de novo;* it cannot be transmitted like a garment; and if it could, it would not fit you, but would attract notice and derision from its uncouthness and incongruity. Hence the earnest and efficient student, whose aim is high and noble, and whose ambition is to relieve suffering humanity and benefit his fellow man, will seek to lay a firm and deep foundation, upon which with each succeeding year of study he may be able to rear a comely superstructure, and at the close of his collegiate career stand up in the conscious assurance of being measurably qualified to do honor to his Alma Mater, and credit to himself in the practice of medicine.

It has been enjoined upon me by the power behind the throne, to present to you some new and striking thoughts—some bright ideas upon this occasion—to go out of the ordinary channel, or rut, of introductory lectures, and mark out a new course for the student, or discourse upon a new theme, which would perhaps interest by its novelty, if it were not of any practical benefit to you. But I do not propose to do anything of the kind. Indeed it is scarcely within the range of possibilities—to say nothing of probabilities—that anything new can be said in the 999th introductory lecture given to the medical schools of our country. "There is nothing new under the sun," was said by the wisest man that ever lived, and I shall not pretend or venture to deny the inspiration that prompted the utterance of the truth. With a disclaimer of making no innovation, it seems to me that some good advice, based on years of observation and a moderate stratum of common sense, may be of service to some of you; that some word may be said which will enable you to commence the study of medicine aright; or, if already somewhat proficient therein, will encourage you to go on till the goal of your ambition is reached. And I know of no way of learning anything, or of being a student, except by patient, diligent application and study. Every student should cultivate and acquire the habit of fixing his mind attentively upon the subject presented him in the lecture-room, or while reading his text-book. There are different degrees of this power in different men. It is not given unto all men, at all times, nor to the same man at all times—the same concentration of thought and energy and action. One man may have, and indeed does have, this power in a greater degree of development than another; he is so constituted by his Creator, with more acute perceptions, and a more retentive memory, and a keener appreciation of the facts and truths of our science; but all possess this power to some extent. Our perceptions may be quickened by observation, and our memory cultivated by experience. It is much more difficult for one man to learn the minutiæ of anatomy than for another; but the latter may not, and frequently does not remember the details of our delicate organization so perfectly as he, who requires a longer time to master it. A fact easily learned is more easily forgotten than is one acquired by hard study. One student at the close of a lecture may be able to detail the important points of it, while at the end of a fortnight his knowledge of it will be much less than is that of another student who has reviewed the points in his own room, and fixed them in his mind, as pegs upon which he can hang somethiug in his future studies. Every student should aim to be *thorough* in acquiring truth, and should learn a subject *once for all*, so that once knowing it he will always know it. Let none of you study a lecture merely for the quiz that should always precede the succeeding lecture, but study each lecture for your whole lives. You will not be able in a single course of lectures to become acquainted with all the subjects presented to you. No one expects this, nor should any one attempt it; if he does, he will signally fail.

Anatomy, Physiology, Chemistry and Materia Medica, constitute the rudimentary branches of the study of medicine, and to these the attention of the student should be first given till a thorough and complete knowledge of them has been acquired. In the first course of lectures the student should devote his time and study to these departments, and to these alone. He may attend the lectures upon Practice, Surgery and Obstetrics, and should remember what he can of them, but his studies should be on the first named. I know full well the dryness of detail attending a thorough study of these branches, and that the practical departments present much greater attraction to the student. But if he does not learn these branches at once, first of all, he will never learn them. Hence my advice to the student on entering an office is to get a set of bones, and "bone in." Acquire a perfect knowledge of each bone, of each process, tubercle and foramen. It is a hard and dry subject, and the temptation will be very great to give it up and take up a book on Practice or Surgery. But let me enjoin upon you to resist such temptations. What would you think of a man who commenced building a house to occupy during his whole life, without first laying a good foundation? You would pronounce him an insane man or a fool. Be careful, my young friends, that you do not pronounce a similar verdict upon yourselves by ignoring the study of the osseous system as the basis of Anatomy, and of Anatomy as the fundamental branch of the whole science of Surgery and Medicine. A thorough and perfect knowledge of it is necessary not only for the surgeon, but also for the successful practitioner of medicine. As a source of mental discipline alone, it is worthy of your earnest attention and study. Hence in most medical schools a preliminary term exists, during which the whole time of the pupil is devoted to this subject alone. The other departments, in consenting to this arrangement, recognize the value of an accurate knowledge of the human system, as it comes from the hand of the Great Architect himself. You have advantages in this respect that the older practitioners of our day did not enjoy. Fifty years ago the facilities for acquiring a knowledge of Anatomy were scarcely available, and the necessity of dissecting as a prerequisite to graduation was established in this country by the Faculty of the Buffalo Medical College, upon its organization twenty-two years ago. Other medical schools at once adopted this provision; and the Legislature of our State, in the law legalizing dissection, to some extent furnishes advantages of *material* which were not afforded in the days of our pupilage. Another very important department, that of Physiology, which has been termed the hand-maid of Anatomy, was elevated to its proper position by the introduction of vivisections within these walls, for the first time in this country. Here Dalton, Flint, Jr., and Mason commenced their career as lecturers and teachers, and nowhere are they excelled, nor in our country even equalled.

It is not necessary for me to give you any hints as to the importance and necessity of prompt and regular attendance upon each and every lecture. You cannot miss a single lecture without breaking a link in the chain; for each succeeding lecture is connected with and dovetailed into the preceding one, so that by losing one, the harmony of the whole is disturbed, and can never be restored. Neither is it necessary for me to invoke your earnest and careful attention to the various subjects, as they are duly presented to you. The best way for you to fix your attention upon a lecture is to take notes of the lecture; by so doing your mind will be fully occupied, and you will have no time to look around the room to see how your fellow is employed. One careless and wayward student, by playfully diverting the attention of the class from the lecture, harms not only himself, but many others also, and he can never repair the injury done to both. The only safe and reliable course for *every* student to pursue is, to be gentlemanly and correct in his deportment at all times, both in and out of the lecture-room. I must crave your indulgence for speaking thus plainly on this subject, for an experience of ten years as a teacher shows me that while the great majority of every class do not need such an admonition, there has been in each some one who ought to have it. Besides grace of manner and an easy deportment are highly essential in your future career as practitioners in medicine. It is a very trite and common saying, "manners make the man;" and of two physicians, he who is the most perfect gentleman—other things being equal—will get the start in the race, and will meet with the earliest success; for more depends upon this than you can now realize.

What has been said respecting the lecture-room here, pertains with equal and even greater force to the wards of the hospitals. By special favor to the Faculty, you are admitted to both the hospitals in our city, and in them, during the term, a great many interesting cases will come up for observation—a careful study of which will be of immense benefit to you in after life. Let me impress upon you the vast importance of making the best use of the advantages afforded you for clinical observation in this College. Do not think for a moment that there are too many students attending the wards for you to be benefited. If you can not learn something in a ward with eighty to one hundred students, what, let me ask, could you expect to learn and observe in a ward with five hundred to one thousand students? And each case, however trifling it may appear to you, is of benefit; for these cases make up the great majority of patients you will meet with in the early part of your professional career. Hence do not ignore what you will find so useful, if properly employed. In the practice of Surgery, you will find the common cases of injury principally engaging your attention; and the successful and skillful management of them will not fail to attract attention to your merits. I know full well how much more attractive to the student a surgical clinic is where one of the capital operations is performed; but these cases are the exception and not the rule in practice. Besides, but few of you will venture to make a capital operation.

Of medical classes in general, not one student in ten makes a surgeon, for the majority have not the opportunity, or if they have, do not improve it. Still there are very many cases which cannot be gotten rid of, and a careful observation of these during your pupilage will amply fit you to attend to them in the best way when you go into business. If opportunity offers, it is well for a student to spend the last of his three years study as *interné* in a hospital, or an alms-house. I shall never forget the advantages I enjoyed in this capacity in the Buffalo Hospital of the Sisters of Charity, and shall always remember with heartfelt gratitude the kindness of my preceptor, Prof. White, in securing for me so desirable a situation, where I was under the immediate instruction of Prof. Austin Flint, Sen., who then had charge of the medical ward, and of Prof. Frank H. Hamilton, then Attending Surgeon of the Hospital. Both are honored names in the annals of the medical department of the University of Buffalo, and in the profession at large. The results of their labors and their efforts are recorded in volumes which take the front rank as text-books in all the medical colleges of our country, and in many in foreign lands. Reference to these men and to the benefits of hospital observation, carries me back twenty years ago, when I made my advent into this city as a medical student. The lectures were then given in what used to be a church edifice, upon the corner of Washington and Seneca streets, where the Post Office now stands. On the third floor of the building, beneath the amphitheatre, was the dissecting-room, bearing no comparison with the commodious rooms of the present edifice. In the basement were stores and shops of various kinds; among them a meat market, where just before Christmas was exhibited a huge, black bear—sleek and fat—ostensibly fed from the butcher's stall, and the proprietor justly anticipated an ample return from the investment on the annual festival of good things—an expectation wholly blasted by the casual remark of a waggish student, that the bear had been fattened by the surplus products of the dissecting-room—instead of fresh meat—and not a pound could the dealer dispose of.

The next year, in October, 1849, these ample rooms were finished, and on the opening of the regular term in November of that year, an introductory lecture was given by Prof. Flint to a very large and intelligent audience, composed of the *elite* of our city, and many ladies graced the occasion with their presence. A large class convened in this very room to listen to his eloquent and interesting lecture, and as I listened with eager ears to the words that fell from his lips, it never for a moment entered my mind that I should ever occupy the place he then occupied, and offer words of cheer and good, cordial greeting to a class of medical students in an introductory lecture. But, gentlemen, we little know what is before us in this world, or the fate that awaits us, in our domestic, public or professional duties. In 1858 I was unexpectedly appointed to the position I have the honor of occupying in this institution. It is a momentous and interesting thought, that I may now be addressing some one, who in a few short years, will fill the place now occupied by some of us.—Time speeds on in rapid, never-wearied flight. He pauses not, but bearing on his wings the seeds of death—the fair, bright things of earth wither as he passeth. A few brief years and the places we now occupy will be filled by others. Study diligently, then, and work faithfully in the discharge of every duty. Let no opportunity for observation or improvement pass by unheeded. Set your aim high, and you will accomplish far more than if you make no aim at all. Study general principles and collect facts; one fact is of far more value than a thousand theories; and facts are what are wanted in the treatment of disease. The importance of a correct diagnosis cannot be too strongly impressed upon your minds. Diagnosis is everything in practice, and he who is skillful in diagnosis will certainly succeed; for with it are combined quick perceptions and good judgment, which, with common sense, will invariably ensure success. There is a certain amount of tact in getting a patient, in the first place, but oftentimes it requires more tact to *keep* a patient than to get one. You must cultivate the *suaviter in modo*, as well as the *fortiter in re.* Be not discouraged if you do not at first meet with that success which you think you deserve. Improve aright the leisure hours of your early professional career, (and you will be likely to have a good many of them,) in copying the notes you take in these rooms for permanent future reference, and in reading and studying too, the works of men who have become eminent in the profession as writers and teachers. Above all, study the writings of him who has been successful in his profession, not only in acquiring a reputation as a writer and teacher, but also in securing that reward for his services which will smooth his declining years. It is too apt to be the case that the best and most active physicians do not record their experience and observation—for want of time—so that very much is lost to the younger members of the profession, to which they have a claim. I care but very little for the opinion of this or that physician or surgeon, unless I know something of him by reputation, or of his superior ability and judgment. You must learn to think and act for yourselves in every case, and not rely upon the opinion of another. It does not necessarily follow that the oldest man in the profession knows the most. He may have had the greatest opportunity for observation, and far the greatest experience; but if he has not kept pace with the advance in medical science, he is but a tyro, compared to what you should be at the close of your collegiate course.

After attending all the lectures of the day at the college, the student needs, and should seek some recreation. He needs some exercise in the open air, and if he does not get this in walking to the college from his boarding place and back again, he should certainly get it from a sense of duty in the preservation of his health. It is not a good plan to board too near the college, for some students do not appreciate the necessity of spending an hour or two out of doors after being confined in the lecture-room all day. After a regular and faithful attendance upon the lectures of the day, two or three hours study in the evening will suffice. From 7½ to 9½ or 10 o'clock in the evening is as much as any student ought to study. Whatever limit you may fix for study, let it be for study in earnest; do not allow any one or anything to interrupt you. Be systematic and regular in your hours of study, and be diligent therein, and you will be surprised to find how much you can learn in a given period. Let perfect silence prevail in your rooms as there is in the best regulated school-rooms, and after due and diligent study for two hours, quiz each other for a little while on the lessons of the day, and then seek that relaxation from study and labor which nature asks before she sends her sweet restorer—balmy sleep. The amount of sleep a man requires depends on circumstances and temperaments. What is one man's food may be another man's poison, so that while six hours may be as much as one student requires, another may not be satisfied with eight. As a general rule, retire early, when you can do so, and after seven or eight hours' repose, rise and study for an hour or two before breakfast. There can be no doubt that one's mind is more active in the morning than after eating. I should add, by way of parenthesis, that this is the theory; I cannot speak of it from any actual experience.

And now, gentlemen, in conclusion, permit me to say that the study and the practice of the science of medicine occupy the highest place and present the greatest claims in all the various pursuits of the human race. Physicians have always been deemed indispensable to the public, and the good physician has always been held in the highest esteem, being, according to the great Grecian poet, "more important than armies to the public good." In ancient times the origin of medicine was referred to the immediate inspiration of the gods, and great and permanent benefits must have sprung from our healing art. Esculapius, with other distinguished physicians, was considered *divine*, and a temple was erected to his memory. Sydenham pronounced "the art of medicine as the best of all worldly gifts, and so much more preferable to all others, as life surpasses all the enjoyments it brings with it." It has always been observed that philosophy and medicine have flourished together, and you need not be informed that great men in every age have devoted their time and energies to its study. Aristotle unfolded the mysteries of our science, and Plato taught the doctrine of the immortality of the soul. The old sage of Cos, Hippocrates, discoursed upon the phenomena of disease. Galen is said to have given the first introductory lecture upon record, and showed that medicine required a superiority of mental over physical power, and also should be placed above all the other arts combined. In every age illustrious names can be found honoring our profession. Haller, Sydenham, Hunter and Rush, names that will never die, showed the high place medicine has always had at the head of human science. The pursuit of this study will not only furnish an ample field for the most disinterested benevolence—the purity of which cannot be questioned—but will also gratify the most aspiring ambition. It is coëxtensive with the whole circle of sciences, and none demands a purer moral character, nor greater mental ability.

Almost every science may be made instrumental in exploring the mysteries of medicine, and each succeeding year reveals more perfectly to our view the intimate connection existing between it and all other sciences. Hence the importance of your acquiring a thorough knowledge of Botany, Mineralogy, Geology, and Chemistry. The physician should have a perfect knowledge of the mental and moral, as well as physical properties of his patient, for he has to deal with all these combined; and it is many times much more difficult to correct aberrations in the former than in the latter. His whole time and all his energy is devoted to the employment of means calculated to save and prolong life, and alleviate human suffering. He is not afraid of the "pestilence that walketh in darkness," nor does he hesitate when called to visit contagious diseases, even of the most horrible and fatal character. His ear is always ready to listen to the appeals of the sick, though degraded and poor; nor does he forsake them while the soul lingers in its mortal tenement. No pursuit furnishes so thorough a moral training from beginning to end; for every day brings with it an opportunity for the gratification of generous impulses and benevolent purposes. Death is no respecter of persons. The knight of the black rod wields his wand over the roof of the splendid mansion, as well as the squalid hovel of want and misery, and at each our skill and sympathy are needed and rendered. It will never be known in this world how much our profession has done for humanity, nor how weighty are its claims upon the grateful and respectful consideration of mankind.

Philosophers have written, and poets sung, denouncing human fame, while every dash of the pen, and every sounding note of the lyre, were but so many efforts to obtain that which they were endeavoring to prove as worthless. You have before you, young gentlemen, the widest field for your ambition. Never in the history of the profession did so fair an opportunity offer for the cultivation of high, honorable and noble principles. We look to you to carry forward and perpetuate the legacy we bequeath you. Those changeless principles that mark out the path to virtue, exert a mightier influence, bind down men's passions more surely, than all the thousand laws and subtleties of legislation, moulded into the strong chain of government. For were they welded together with whatsoever strength its links would be riven and melted by the lightning strokes of passion. Indeed it is the true end of government, rather to develop these virtuous springs of action, and to thus bring man to truth and justice than to take revenge for its violated majesty. With these principles as your beacon-light, drawing you on to the attainment of all that is just and honorable and true in the profession of your choice, let me urge you to go forward, and add to the scope of human knowledge already acquired, for the benefit of humanity and the race at large. Let "no pent-up Utica contract your powers,' but mindful of your duty to yourselves, the profession and the world, so live that when the summons comes to draw you hence, you can pass away as serenely,

"As one who wraps the drapery of his couch
About him, and lies down to pleasant dreams."

